# Prenatal Diagnosis and Prognosis of Abdominal Arteriovenous Fistulae: A Comprehensive Case Series and Systematic Review

**DOI:** 10.3390/diagnostics14080826

**Published:** 2024-04-17

**Authors:** Anda Ungureanu, Rodica Daniela Nagy, Cristian Constantin, Ioana Andreea Gheonea, Dominic Gabriel Iliescu

**Affiliations:** 1 Doctoral School, University of Medicine and Pharmacy of Craiova, 200349 Craiova, Romania; ungureanu_anda@yahoo.com (A.U.); dominic.iliescu@yahoo.com (D.G.I.); 2 Department of Cardiology, University Emergency County Hospital Craiova, 200642 Craiova, Romania; 3 Ginecho Clinic, Medgin SRL, 200333 Craiova, Romania; 4 Department of Obstetrics and Gynecology, University Emergency County Hospital Craiova, 200642 Craiova, Romania; 5 Department of Radiology and Medical Imaging, University of Medicine and Pharmacy of Craiova, 200349 Craiova, Romania; cristian.constantin2364@gmail.com (C.C.); iagheonea@gmail.com (I.A.G.); 6 Department of Obstetrics and Gynecology, University of Medicine and Pharmacy of Craiova, 200349 Craiova, Romania

**Keywords:** intrahepatic fistulae, outcome, prenatal diagnosis, ultrasound

## Abstract

This study had two main objectives. Firstly, we conducted a thorough literature review on the prenatal diagnosis of abdominal congenital arteriovenous fistulas (CAVFs) involving the abdominal aorta and hepatic arteries. Secondly, we aimed to provide detailed descriptions of eight additional cases diagnosed at our medical center and assess the outcome of this anomaly for informed counseling. We conducted a systematic search of online databases using specific keywords like “outcome”, “ultrasound”, “intrahepatic fistulae”, and “fetal venous anomalies”, focusing on studies published between 1998 and 2023. We selected 10 relevant articles and analyzed 13 cases. Additionally, we conducted a five-year prospective study in two referral centers, identifying eight CAVF cases with an incidence rate of 0.16%. Among the 21 cases evaluated, 11 resulted in live births, all of which received treatment. However, four cases (36.3%) had poor postnatal outcomes and neonatal demise due to heart failure. Prenatal signs of poor fetal hemodynamics, including cardiomegaly or hydrops, were observed in 52.3% of cases, regardless of outcome. Our findings highlight the rarity of this vascular malformation and emphasize the importance of effective treatment to avoid unfavorable outcomes. The long-term effectiveness of prenatal treatment or postnatal embolization remains uncertain, with liver transplantation being considered the most reliable treatment option.

## 1. Introduction

Congenital abdominal arteriovenous fistulas (CAVFs) are uncommon vascular anomalies that typically arise during the embryonic period between 4 and 10 weeks of gestation. They are characterized by abnormal connections between an artery and either portal or hepatic veins, with an estimated incidence of approximately 1 in 100,000 live births, indicating their rarity in the general population [1]. However, their true prevalence and clinical manifestations are often underestimated due to underdiagnosis [1]. This can be attributed to undetected cases or those misdiagnosed as other vascular malformations. The clinical presentation of CAVFs can vary; some cases are asymptomatic, while others exhibit symptoms before and after birth [2]. The manifestation of symptoms depends on the type of shunt present and the extent to which the arterial and venous flows are altered. Histologically, CAVFs are characterized by the presence of dysplastic arteries and veins. Unlike hepatic hemangiomas, they are not associated with soft-tissue masses. Instead, these abnormal connections bypass the tissue capillary bed, leading to potential complications, such as heart failure, anemia, embolism, and bleeding. Dilated arteries ensure the nourishment of these fistulas, while dilated veins facilitate drainage [2]. In addition, the differential diagnosis of hepatic vascular lesions includes umbilical–portal–systemic venous shunts, defined as abnormal venous system communications between the umbilical, portal, and DV (ductus venosus) systems [3]. These venous shunts are frequently well tolerated and often regress after birth, although some types have been associated with fetal anomalies and intrauterine growth restriction [4].

The inherent course of these anomalies tends to progress towards deterioration and, ultimately, fetal demise, thereby involving significant implications in both the prenatal and postnatal periods. Early neonatal clinical manifestations are closely linked to a strikingly high mortality rate ranging from 50% to 90%. These abnormalities can affect the liver. They are conventionally associated with cavernous lymphangioma, hemorrhagic telangiectasia (Osler–Weber–Rendu disease), and trisomy 21 [1,5,6]. Doppler ultrasound has been a valuable diagnostic tool for identifying these fistulas, providing essential information on the flow direction and velocity type (i.e., arterial, portal, or hepatic) [4].

Recent recommendations for fetal echocardiographic assessment, in line with major guidelines, focus on a range of technical and procedural factors. These include the use of higher-frequency transducers for more accurate detection of subtle defects, with recognition of the necessary balance between acoustic penetration and resolution. Grayscale images are essential, with system settings emphasizing high frame rate, increased contrast, and high resolution. Utilizing cine-loop feature and image storage is recommended for real-time assessment of cardiac structures and anomaly identification. The examination should be recorded in a manner that allows for later review and ensures proper patient identification and image labeling. These recommendations contribute to more precise evaluation and appropriate diagnosis of fetal cardiac malformations, ensuring clinical practice aligns with current standards [7]. Additionally, it is important to note that in cases of suspected fetal arteriovenous fistula, extended fetal echocardiography is recommended as summarized in the reference cited. Moreover, indirect signs of such a fistula may be detected during fetal echocardiography performed for other indications, and echocardiography specialists should be aware of these markers, such as cardiomegaly or venous vessel dilatation. Implementing these practices into clinical routine can contribute to comprehensive evaluation and early identification of fetal cardiac anomalies.

The ubiquitous adoption of ultrasound imaging and advancements in diagnostic methodologies have significantly increased the rate of detection of anomalies encompassing vascular malformations among prenatal and postnatal populations. The main objective of this comprehensive review was to gain insights into the prognosis of abdominal arteriovenous fistulas. This understanding was aimed at facilitating well-informed and thorough parental counseling. By understanding the short- and long-term prognostic implications of this vascular anomaly, parents can be appropriately counseled to make well-informed decisions regarding their children’s healthcare. Moreover, an in-depth understanding of the pathophysiology and clinical course of arteriovenous fistulas has the potential to reveal opportunities for effective interventions. Our objective was to conduct a comprehensive literature review regarding the prenatal diagnosis of CAVFs, including data from patients diagnosed at our medical center. The outcome of these cases could facilitate informed and appropriate parental counseling. By identifying viable therapeutic approaches and management strategies, healthcare providers can strive to optimize patient outcome and improve the overall prognosis of patients affected by this condition.

## 2. Materials and Methods

Using an objective approach, we conducted an exhaustive search of PubMed and Scopus, using specific keywords, including “outcome”, “ultrasound”, “intrahepatic fistulae”, and “fetal venous anomalies”. The literature search was limited to studies published between 1998 and 2023. Subsequently, two independent evaluators reviewed the initial findings against the predefined inclusion criteria.

This study also presents the clinical features of eight cases diagnosed at our tertiary center, the Prenatal Unit of University Emergency County Hospital Craiova, during a prospective study spanning five years, from February 2019 to March 2023. The study was approved by the Institutional Ethics Committee prior to its initiation. During the study, a systematic color Doppler assessment of the abdominal vascular system was conducted for all pregnant women seeking prenatal care at our center, including routine examinations and referred cases. The gestational age range was between 13 and 30 weeks of gestation. Transabdominal ultrasound (US) examinations were performed using Voluson E10 and E8 ultrasound machines (GE Medical Systems) equipped with a 4–8-MHz curvilinear transducer. Implementing the As Low As Reasonably Achievable (ALARA) principle ensured that the mechanical and thermal indices remained at their lowest feasible levels during color Doppler assessment. The diagnosis criteria for CAVFs encompass identifying an atypical vascular area supplied by an artery and drained by the venous system, typically portal or hepatic. Additionally, the presence of a pulsatile arterial flow at the connecting vessels, along with cardiomegaly and dilation of portal and/or hepatic veins, serves as markers of the steal phenomenon from arterial to venous compartments. If abnormal scan findings were identified, a second opinion was sought through evaluation by two experienced examiners with five years of experience. Following confirmation and interdisciplinary communication, the women were informed about the long-term prognosis of their cases and presented management options. In addition to the fetal medicine specialist, the counseling team involved in these discussions included a geneticist, a neonatal pediatrician, and a pediatric surgeon. Written informed consent was obtained from all participants before ultrasound examinations.

The ethical aspects of the study were addressed and approval was granted by the Ethics and University and Scientific Deontology Commission of the University of Medicine and Pharmacy Craiova, Romania. This endorsement confirms that the study adhered to the ethical principles outlined in the University Code of Ethics and the Declaration of Helsinki, ensuring the proper conduct of research throughout the investigation. 

## 3. Results

### 3.1. Results of the Systematic Review

The initial search identified 128 articles, of which 15 duplicates were excluded. The selection was refined to 25 articles featuring abstracts and titles relevant to our research. Subsequently, a thorough evaluation of the full content of these articles resulted in the inclusion of 11 papers for this review. One non-English article was excluded. Of the 10 articles finally included in this systematic review, 7 were case reports and 3 were case series, encompassing a total of 13 patients whose prenatal and postnatal outcomes were evaluated. All patients were successfully diagnosed prenatally using ultrasonography. The gestational age at diagnosis ranged from 14–36 weeks. The median gestational age at diagnosis, calculated from the available data, was found to be 26 weeks. The anomaly was tortuous vascular vessels in the fetal liver, characterized by an arterial connection to a fistulous venous drainage system within the liver. The findings of the included studies are summarized in Table 1.

The prenatal diagnosis of an intrahepatic arteriovenous fistula should meet several criteria, including an atypical vascular area fed by an artery and drained by the venous system (portal or hepatic). Prenatal findings include a pulsatile arterial flow waveform at the level of the connecting vessels, cardiomegaly, and dilatation of the portal and/or hepatic veins as markers of the steal phenomenon from the arterial to the venous compartments. As more blood is shunted through this drainage system, fetal cardiac output is increased due to the steal phenomenon, a process that can result in high-output heart failure or hydrops [8].

Interestingly, complex cases have been previously reported. Tseng et al. reported a case with two branches of the hepatic artery that drained into the portal vein and communication between a branch of the hepatic artery and a branch of the middle hepatic vein [9]. An essential aspect of the reported case is that a patent ductus venosus may divert the portal vein blood into the inferior vena cava with a delayed postnatal clinical manifestation.

Some studies have highlighted the need for a differential diagnosis between hepatic hemangiomas and arteriovenous malformations. In addition to the abovementioned features, high-output cardiac failure is more common in CAVFs, which never regress spontaneously. Hemangiomas are benign tumors with rapid growth prenatally or in the first year of life, but with regression in the subsequent years [10,11,12].

Two cases of arteriovenous malformations involving the abdominal aorta and umbilical vein have been reported, all presenting with a genetic anomaly, trisomy 21 [1]. The most frequently associated anomalies were signs of poor fetal hemodynamics, including cardiomegaly, hydrops, and pleural effusion.

#### 3.1.1. Postnatal Manifestations

The postnatal clinical manifestations depend on the type of shunt (hepatohepatic or hepatoportal). Both types have hemodynamic consequences, and the prognosis worsens if clinical manifestations occur early in postnatal life. Hepatohepatic shunts are associated with congestive heart failure, while hepatoportal shunts lead to portal hypertension with thrombocytopenia secondary to local platelet sequestration, described as consumptive coagulopathy or Kasabach–Merritt syndrome [13,14].

#### 3.1.2. Prenatal and Postnatal Management

Medical treatment options should be considered for symptomatic prenatal cases. In 1995, Mejides et al. [11]. reported the first case of prenatal diagnosis and treatment of an arteriovenous fistula. Prenatal management was initiated using the intrauterine application of hydrocortisone to the umbilical vein and amniotic fluid, coupled with maternal digoxin, which resulted in decreased liver vascularity, increased hematocrit and platelet counts, and improvement in the fetal echocardiogram. Postpartum steroid treatment was postponed until the 18th day, when the neonate developed signs of heart failure. Demirci et al. [2]. reported the first prenatal case of a hepatic arteriovenous fistula that was successfully treated with steroids and propranolol until term. Both studies suggest that propranolol has a more favorable side effect profile than steroids, making it a more reliable option for prenatal treatment in symptomatic cases involving arteriovenous fistulas.

Favorable postnatal outcome may be predicted in the absence of clinical manifestations; however, close follow-ups for portal hypertension or cardiac failure are recommended. Ligature of the left hepatic vein at six months of age may be necessary because of the development of shortness of breath, malaise, poor appetite, and watery diarrhea. The authors noted a good outcome at the 3-year follow-up [9].

Lima et al. reported two cases of hepatohepatic shunts, showing that the postnatal management of arteriovenous malformations depends on the case particularities and should not be universal. They described different therapies, such as embolization, which require the fistula to be precisely located and all feeding arteries to be completely occluded as close as possible to the lesion. Partial hepatic resection is recommended exclusively for well-defined, localized lesions that are not associated with hemodynamic complications [10]. 

Embolization should be performed in selected cases. Reports by Lima et al. and Botha et al. suggest that embolization is effective in cases with a single feeder arterial vessel, as recanalization may occur when there is a more complex anomaly [10,12].

Hepatic resection is the treatment of choice for localized anomalies [10,12]. One case reported by Lima et al. and another reported by Gedikbasi et al. had no prenatal or postnatal signs of heart failure or hydrops. However, embolization was not performed because of the involvement of large feeding arteries. Hepatic resection was performed, with good outcome [10,14,15].

In the cases reviewed, various diagnostic findings were observed, including vascular anomalies such as hypoechogenic structures in the liver, prominent portal and hepatic veins, dilated vascular channels, and tufts of vessels. These anomalies were often associated with fetal cardiomegaly, polyhydramnios, and pericardial effusion. The types of fistulas identified included hepatic artery-portal vein, hepatic artery-hepatic vein, and hepatic artery-umbilical vein connections. Spectral analyses were utilized to confirm arterial flow in many cases. Prenatal management strategies varied and included intrauterine corticosteroid therapy, maternal digoxin administration, and prophylactic use of steroids and propranolol. Postnatally, treatments ranged from embolization to hepatectomy, depending on the specific characteristics of the anomaly. The outcomes of these interventions varied, with some cases experiencing stable conditions and favorable outcomes, while others unfortunately resulted in neonatal death due to cardiac failure. Overall, the diagnostic modalities employed, the types of anomalies identified, and the treatments administered demonstrate the complexity and heterogeneity of intrahepatic arteriovenous fistulas in the reviewed cases.

### 3.2. Case Series

Throughout the study period at our institution, comprehensive ultrasound evaluations of the abdominal vasculature was performed in 5,340 pregnancies. Within this cohort, eight cases of CAVFs were identified, leading to an incidence rate of 0.16%. The prenatal findings and subsequent outcome of the eight CAVF cases are presented in Table 2.

Among the eight CAVF cases studied, six (87.5%) were associated with other anomalies. In five of the cases (62.5%), signs of compromised fetal hemodynamics were observed. Furthermore, this investigation revealed the presence of vascular, cerebral, and skeletal malformations previously associated with CAVFs. Genetic testing was performed in six cases, yielding abnormal results in four (66.6%); the specific genetic aberrations identified, included trisomy 21, trisomy 18, monosomy, and mosaicism 17—a case already published [2]. Spectral analyses revealed an arterial flow in all evaluated cases.

Prenatal management primarily comprised regular follow-ups and detailed anatomical and genetic evaluations. In the postnatal period, active management was feasible in only one case; the remaining cases resulted in abortion or intrauterine fetal demise.

In the following section, we present the characteristics of eight cases and highlight their diagnostic criteria and management characteristics.

**Figure 1:**
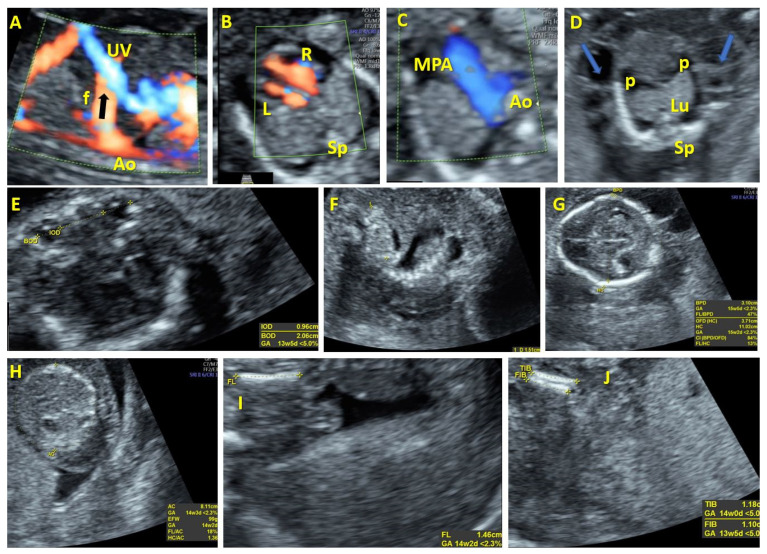
Case 1. Aorto-umbilical arteriovenous fistula at 17 weeks of gestation. (**A**): Color Doppler evaluation in the sagittal plane of the fetal abdomen showing abnormal vascular communication between the aorta and intrahepatic umbilical vein. (**B**): Color Doppler evaluation in the transverse plane of the fetal heart showing the enlarged right heart. (**C**): Three vessel view. (**D**): Transverse plane of the fetal thorax showing generalized edema and pleurisy. (**E**): Sonographic image showing hypotelorism. (**F**): Sonographic image showing dysplastic kidneys. (**G**–**J**): Fetal biometry demonstrates early IUGR (intrauterine growth restriction). UV—umbilical vein. MPA—main pulmonary artery. Ao—aorta. Sp—spine. R—right. L—left. p—pleurisy. Lu—lungs. f—fistulous communication.

Case 1 involved a 26-year-old pregnant woman who was referred to our institution at 17 weeks of gestation for an abnormal vascular formation in the abdomen. A detailed evaluation showed a significant connection between the initial segment of the intrahepatic umbilical vein and the abdominal aorta, resulting in significant hemodynamic consequences and ultimately leading to the development of fetal hydrops. A pulsatile arterial flow waveform was observed at the connecting vessel. Following genetic testing, Turner syndrome was diagnosed. Additional sonographic anomalies were noted, including an enlarged right heart, early intrauterine IUGR (intrauterine growth restriction), hypotelorism, and dysplastic kidneys (Figure 1). The couple chose termination of the pregnancy (TOP). 

Similar to Case 1, Case 2 involves a pregnant woman who presented with an abnormal vascular formation in the abdomen during prenatal evaluation at 19 weeks of gestation. Detailed ultrasound examination revealed an intrahepatic arteriovenous complex malformation characterized by significant connections between the initial segment of the intrahepatic umbilical vein and the abdominal aorta. This complex malformation led to hemodynamic consequences, resulting in fetal distress. The ultrasound findings also indicated additional sonographic anomalies, including vermian malrotation, partial agenesis of the corpus callosum, frontal bossing, and a low nasal bridge, suggestive of structural abnormalities. Moreover, dilation of the right heart chambers was observed, further indicating the severity of the fetal condition. Genetic analysis revealed mosaic trisomy 17, highlighting the genetic component associated with the anomalies. Due to the unfavorable fetal prognosis and the presence of multiple anomalies, the couple chose termination of the pregnancy at 22 weeks of gestation.

Case 3 involves a pregnant woman who presented with an atypical vessel anomaly detected during prenatal evaluation at 22 weeks of gestation. Ultrasound examination revealed an abnormal vascular connection between the abdominal aorta and umbilical vein, suggestive of an arteriovenous malformation. This anomaly was accompanied by additional fetal anomalies, including hydrocephalus and corpus callosum hypoplasia, indicative of central nervous system abnormalities. Despite the presence of these structural anomalies, spectral analyses confirmed arterial flow within the anomalous vessels. Throughout the follow-up period, the fetal condition remained stationary, with no significant changes observed. However, due to the severity of the fetal anomalies and the unfavorable prognosis, the couple opted for termination of the pregnancy at 23 weeks of gestation. 

Case 4 involves a pregnant woman who underwent prenatal evaluation at 13 weeks of gestation, revealing an atypical vessel anomaly characterized by an abnormal connection between the abdominal aorta and umbilical vein. This anomaly was accompanied by the presence of cystic hygroma, and portal venous system agenesis (PVSA), indicative of additional fetal vascular abnormalities. Spectral analyses confirmed arterial flow within the anomalous vessels. Although follow-up assessments were planned, they were not performed due to the onset of fetal distress, reflecting compromised fetal well-being. Consequently, the pregnancy was terminated.

In Case 5, a pregnant woman at 13 weeks of gestation underwent prenatal assessment revealing an atypical vessel anomaly characterized by an abnormal connection between the aorta and hepatic vein. This anomaly was accompanied by bilateral radius agenesis. Spectral analyses to confirm arterial flow were not conducted. Follow-up evaluations were conducted, revealing a stable fetal condition; however, subsequent genetic testing identified trisomy 18 (T18), a chromosomal abnormality associated with developmental delays and multiple congenital anomalies. Due to the severe fetal abnormalities and the prognosis associated with T18, the pregnancy was terminated.

In Case 6, a pregnant woman presented at 13 weeks of gestation with prenatal ultrasound revealed an atypical vessel anomaly characterized by an abnormal connection between the aorta and umbilical vein. This anomaly was accompanied by multiple fetal abnormalities, including megacystis, hand and foot malposition, skeletal malformations, hygroma, hemivertebra, ventricular septal defect, and absent cavum septum pellucidum. Further diagnostic evaluations, such as spectral analyses to confirm arterial flow, were not conducted. The pregnancy was terminated due to the poor prognosis associated with the fetal abnormalities.

**Figure 2:**
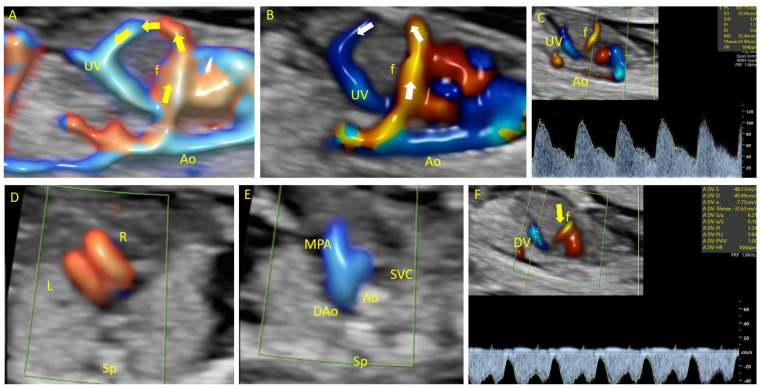
Case 7. Aorto-umbilical arteriovenous fistula at 13 weeks of gestation. (**A**,**B**): Color Doppler evaluation in the sagittal plane of the fetal abdomen shows abnormal vascular communication between the aorta and intrahepatic umbilical vein. (**C**): Color Doppler evaluation in the sagittal plane of the fetal abdomen shows a pulsatile arterial flow waveform at the fistulous vessel. (**D**): Color Doppler evaluation in the transverse plane of the fetal heart shows normal fetal heart anatomy. (**E**): Three vessel view. (**F**): Sagittal plane of the fetal abdomen showing a typical waveform at the ductus venosus. UV—umbilical vein. MPA—main pulmonary artery. Ao—aorta. Sp—spine. R—right. L—left. f—fistulous communication. SVC—superior vena cava. DAo—descending aorta. DV—ductus venosus.

In Case 7, a 27-year-old woman at 13 weeks of gestation with a twin pregnancy presented at our center for an anomaly scan. An intrahepatic arteriovenous malformation (Figure 2) was identified during fetal scanning. The malformation involved an atypical vascular connection between the abdominal aorta and umbilical vein (UV). Pulsatile arterial flow with a high-peak-velocity fistula was observed. Genetic analysis of the affected fetus revealed the presence of trisomy 21. Despite the genetic abnormality, no other sonographic anomalies were detected. Intrauterine demise of the affected fetus occurred at 15 weeks of gestation. The remaining fetus had a normal outcome. 

In case 8, the patient underwent an interdisciplinary examination at the County Emergency Clinical Hospital in Craiova involving the obstetrics, gynecology, pediatric cardiology, and radiology imaging departments. A fetal vascular malformation, an aorto-hepatic arteriovenous fistula in the form of an abnormal vascular communication between the aorta and right hepatic vein, was diagnosed at 27 weeks of gestation. The result was dilation of the hepatic vein and its branches due to additional vascular flow from the aorta through the aforementioned fistulous communication. No further anomalies were identified; however, right ventricular overload and dilatation of the right heart were observed due to the vascular steal phenomenon, characteristic of fistulas. No signs of fetal compromise such as hydrops, were observed. Considering the age of the pregnancy and the nature of the anomaly, close monitoring of the pregnancy was recommended with additional ultrasound examinations beyond the standard evaluation by a multidisciplinary team involving a pediatric cardiology specialist. A premature neonate with low Apgar score was delivered via cesarean section at 33 weeks of gestation because of fetal distress. The patient required resuscitation and orotracheal intubation immediately after birth. A subsequent postnatal evaluation revealed generalized edema, severe heart failure, tricuspid insufficiency, and pulmonary hypertension. The child’s condition remained critical and was marked by the absence of diuresis. Embolization of the fistulous tract was performed, while the hemorrhagic syndrome required transfusions. The newborn’s general condition was complicated with hepatorenal insufficiency, severe heart failure (NYHA class IV), severe right ventricular dysfunction, and anuria. The newborn died two days after birth due to cardiorespiratory arrest.

The treatment options for CAVFs discussed in the article include:

Prenatal Medical Therapy: Prenatal management involves the administration of medical therapy to the fetus while still in the womb. This may include the intrauterine application of medications such as hydrocortisone to the umbilical vein and amniotic fluid, along with maternal digoxin. Prenatal medical therapy aims to reduce liver vascularity, improve hematocrit and platelet counts, and mitigate the adverse effects of fetal cardiac overload. Studies have suggested that medications like propranolol may have a more favorable side effect profile than steroids for prenatal treatment.

Postnatal Embolization: Postnatal embolization involves the occlusion of abnormal blood vessels feeding the arteriovenous fistula. This procedure aims to redirect blood flow away from the abnormal vascular malformation, thereby reducing the risk of complications such as congestive heart failure. However, embolization may have associated risks, including rapid collateral vessel formation, hepatic necrosis, or abscess formation. It is recommended in carefully selected cases, particularly those with a single feeder arterial vessel, to mitigate the risk of recanalization.

Hepatic Artery Ligation: Hepatic artery ligation involves the surgical ligation or closure of the hepatic artery, which supplies blood to the liver. This procedure aims to restrict blood flow to the arteriovenous fistula, thereby reducing the risk of complications such as congestive heart failure or portal hypertension. However, hepatic artery ligation may also have associated risks, including hepatic necrosis or abscess formation. It is considered as a treatment option, especially in cases where embolization is not feasible.

Partial Hepatic Resection: Partial hepatic resection involves the surgical removal of a portion of the liver affected by the arteriovenous fistula. This procedure aims to excise the abnormal vascular malformation, thereby restoring normal liver function and mitigating the risk of complications. Partial hepatic resection is recommended for well-defined, localized lesions that are not associated with significant hemodynamic alterations. It is associated with favorable outcomes, particularly in cases where embolization or ligation are not feasible.

Liver Transplantation: Liver transplantation may be considered in severe cases of intrahepatic arteriovenous fistulas where other treatment modalities have failed or are not feasible. This procedure involves the surgical removal of the diseased liver and its replacement with a healthy donor liver. Liver transplantation aims to restore normal liver function and mitigate the risk of complications associated with the arteriovenous fistula. However, it is considered a more invasive and high-risk treatment option, typically reserved for cases where other options have been exhausted.

Overall, the choice of treatment modality depends on various factors, including the severity of the arteriovenous fistula, the presence of associated complications, and the patient’s overall health status. Treatment decisions should be made in consultation with a multidisciplinary team of healthcare professionals to ensure the best possible outcome for the patient.

## 4. Discussions

CAVFs are rarely isolated and usually associated with cardiac dysfunction and structural malformations. In our case series, the occurrence rate of CAVFs was 0.16%, and we observed an overall unfavorable outcome, with six of the eight cases resulting in abortion (75%) and one case ending in fetal demise. In the live birth case, embolization was performed. Unfortunately, neonatal death occurred due to cardiac failure. Regarding the cases from the analyzed studies, the prognosis was generally favorable when treatment was applied. Of 13 cases reported in the literature, 10 patients underwent postnatal treatment. Seven of the ten patients (70%) had a good prognosis. Of these seven patients, four underwent hepatectomy (57.1%); in one case, only steroids and diuretics were administered; in one case, only propranolol was administered; and in one case, the patient underwent ligature of the left hepatic vein. Of the ten cases that received postnatal treatment, three underwent embolization, all resulting in neonatal death due to cardiac failure. 

In addition, arterio-venous fistulas are likely to induce high-output cardiac failure, with an increased mortality rate of 50–90% [16]. The steal phenomenon was present in 14 of 21 (66%) total cases (reviewed and our cases), and the flow waveforms at the abnormal fistulous connections had a pulsatile pattern. Of the 21 cases, 11 live births occurred, and all underwent treatment. Of the 11 cases, 4 (36.3%) presented with poor postnatal outcome and neonatal demise due to heart failure. All four cases were treated with embolization. Both cases with a favorable short-term outcome and those leading to abortion showed prenatal signs of compromised fetal hemodynamics, such as cardiomegaly or hydrops (52.3%). Furthermore, these indicators were present in both groups, with the good outcome group also displaying such signs. In addition to heart failure, the steal phenomenon may cause microangiopathic hemolytic anemia, thrombocytopenia, and Kasabach–Merritt syndrome consumptive coagulopathy, which also occurred in our case 8 [17].

Chromosomal abnormalities have been frequently associated with vascular anomalies, with 15% of trisomy 21 cases associated with vascular malformations [6,8]. Of the 21 patients included in this study, 12 underwent karyotyping. Abnormal genetic results were present in six cases (50%): three trisomy 21 cases, one X monosomy case, and one mosaic trisomy 17 case [1,18]. After analyzing all the reports in our review, we could not draw conclusions regarding the genetic assessment. However, there is an increased risk of aneuploidy in the presence of vascular abnormalities [6].

As intrahepatic arteriovenous fistulas do not regress spontaneously, various treatments have been proposed, such as prenatal medical therapy, postnatal embolization, hepatic artery ligation, laser therapy, hepatic resection, and liver transplantation [10,19]. 

In the presence of cardiac failure in preterm pregnancies, intrauterine corticosteroid and propranolol therapy may improve the outcome [2,11]. This therapy was used with encouraging results in two cases of arteriovenous fistulae. Although the delivery in one case was at 31 weeks of gestation, the baby had no signs of postpartum heart failure, and no treatment was administered after birth. As the postpartum status changed on the 18th day by fetal tachycardia, tachypnea, and increased hepatic vascularity, the decision was to administer steroids and diuretics, with significant improvement after one week. The applicability of steroid and/or propranolol therapy for the prenatal management of arteriovenous fistulas is controversial, and further studies are required [2].

Postnatal embolization and hepatic artery ligation have increased morbidity and mortality rates due to complications such as rapid collateral vessel formation, hepatic necrosis, or abscess formation [20]. In this review, four cases that underwent embolization as postnatal management had poor outcome, as congestive heart failure occurred. This management approach requires sonographic follow-up because of the potential recanalization of vascular anomalies [12].

Partial hepatic resection is the optimal surgical treatment [21]. This procedure is recommended for well-defined localized lesions with no signs of postnatal hemodynamic alterations. Although long-term follow-up was only possible for a maximum of 3 years, the best outcome was observed in surgically treated cases.

## 5. Conclusions

Intrahepatic arteriovenous fistulas are rare vascular anomalies that significantly affect prenatal fetal hemodynamics, as they are frequently associated with cardiomegaly, cardiac failure, hydrops, and structural and chromosomal abnormalities. These anomalies may cause postnatal portal hypertension and high-outflow cardiac failure.

Studies suggest that propranolol demonstrates a favorable side effect profile compared with steroids, and is thus a more dependable prenatal treatment option in symptomatic cases involving arteriovenous fistulas. In cases with no apparent postnatal clinical manifestations, more favorable outcome may be possible. Closely monitoring these individuals for the development of portal hypertension or cardiac failure is crucial. 

Embolization is recommended in carefully selected cases. Evidence suggests that embolization is particularly effective when dealing with cases characterized by a single-feeder arterial vessel, as it may mitigate the risk of recanalization, which could be more probable in more complex vascular anomalies.

Hepatic resection is the therapeutic option with the best outcome for managing localized anomalies. In instances where embolization was not performed due to the involvement of large feeder arteries, no prenatal or postnatal signs of heart failure or hydrops were evident. Consequently, hepatic resections were performed in patients presenting with localized anomalies with favorable outcome. Surgical treatment is associated with the best outcome.

Prenatal diagnosis and postnatal confirmation are essential for monitoring and therapy before the development of complications, which may affect long-term outcome.

In such cases, parents should be counseled and informed about the impaired liver function arising from this fetal venous abnormality and the prenatal and postnatal treatment options.

## Figures and Tables

**Table 1:** Congenital abdominal arteriovenous fistula cases retrieved during the systematic review.

References	GA	Vascular Anomaly	Associated Anomalies	Type of Fistula	Spectral Analyses	Prenatal Management	Prenatal Outcome	Karyotype	Postnatal Management	Postnatal Outcome
Jouannic et al. [8] 1998	30 WG	Vascular hypoechogenic structure in the liver	None	Porto-hepatic	Not evaluated	Follow-up	Stationary	Not evaluated	Embolization	Death due to cardiac failure
Tseng et al. [9] 2000	35 WG	Prominent portal and hepatic veins	Oligohydramnios cardiomegaly	Hepatic artery-portal vein-middle hepatic vein	Arterial flow	Follow-up	Atable	Not evaluated	Ligature of the left hepatic vein	Patent ductus venosus at 6 months, good at 3 years old
Lima et al. [10] 2005	25 WG	Suprarenal aortic and left hepatic artery dilatation	Mild cardiomegaly Minimal pericardial effusion	Hepatic artery-hepatic vein	Not evaluated	Follow-up	Stable	Normal	Embolization	Death due to cardiac failure
Lima et al. [10] 2005	27 WG	Fluid-filled area affecting the upper part of the left hemi abdomen	Severe cardiomegaly	Hepatic artery-hepatic vein	Arterial flow	Follow-up	Stable	Normal	Left hepatectomy	Good at 20 weeks postnatal
Mejides et al. [11] 1995	29 WG	Anechoic areas within the fetal liver	Cardiomegaly Cardiac failure Moderate pericardial effusion	Hepatic artery-hepatic vein	Arterial flow	Intrauterine corticosteroid therapy Maternal digoxin	Stable	Normal	Prednisone and furosemide	Good
Botha et al. [12] 2004	34 WG	Echogenic dilated vascular channels within the liver	Cardiomegaly polyhydramnios	Hepatic vein-hepatic artery-right and left internal mammary artery-	Not evaluated	Follow-up	Fetal distress	-	Embolization	Death due to cardiac failure
Douhnai et al. [13] 2018	26 WG	A tuft of vessel in the left lobe, enlarged left hepatic artery, enlarged left portal vein	Polyhydramnios	Hepatic artery-portal vein	Arterial flow	Follow-up	Stable	-	Follow-up	Good Patent DV
Gedikbasi et al. [14] 2008	36 WG	Vascular structure/lake	None	Hepatic artery-hepatic vein-superior mesenteric artery	Arterial flow	Follow-up	Stable	-	Right extended hepatectomy, cholecystectomy, local removal of the vascular ectasia/ structure	Good
Demirci and Celayir [2] 2020	32 WG	Thick multiseptic cystic lobulated contoured mass	None	Hepatic artery-hepatic vein-umbilical vein	Not evaluated	Follow-up	Stable	Not evaluated	Prophylaxis with steroids and Propranolol, followed by right extended hepatectomy	Good at 18 months
Demirci and Celayir [2] 2020	24 WG	Large celiac artery, enlarged hepatic veins.	Ascites Dilated cardiomegaly	Hepatic artery- hepatic vein	Normal venous pattern	Dexamethasone propranolol	Stable under treatment	Not evaluated	Propranolol	Good at 2 months
Hartung et al. [1] 2000	14 WG	Atypical vessel	Hydrops Complete atrioventricular canal	Abdominal aorta-umbilical vein	Arterial flow	Follow-up	Stationary	Trisomy 21	Not applicable	Abortion at 15 WG
Hartung et al. [1] 2000	31 WG	Dilated umbilical vein joined by hepatic artery	Hydrops Polyhydramnios, suspicious for duodenal atresia	Hepatic artery-umbilical vein	Arterial flow	None	No follow-up	Trisomy 21	Not applicable	Stillbirth at 36 WG
Zhou et al. [15] 2016	31 WG	Dilated umbilical vein, vascular structure in the middle of the liver	Cardiomegaly Perimembranous ventricular septal defect, left-sided inferior vena cava	Aorta-portal vein-umbilical vein	Arterial flow	Follow-up	Stable	Normal	Extended right hepatectomy	Good at 3 months

WG, weeks of gestation; DV, ductus venosus.

**Table 2:** Prenatal cases with arteriovenous malformations diagnosed at our center—perinatal management and outcome.

Case nr.	GA	Vascular Anomaly	Associated Anomalies	Type of Fistula	Spectral Analyses	Prenatal Management	Prenatal Outcome	Karyotype	Postnatal Management	Postnatal Outcome
1	17	Atypical vessel	Dilation of right heart chambers, early IUGR, hypotelorism, dysplastic kidneys, hydrops fetalis	Aorta-umbilical vein	Arterial flow	Follow-up	Fetal distress	45X	Not applicable	Abortion at 19 WG
2	19	Intrahepatic arteriovenous complex malformation	Vermian malrotation, partial agenesis of the corpus callosum, frontal bossing, low nasal bridge, dilation of right heart chambers	Aorta-umbilical vein	Arterial flow	Follow-up	Fetal distress	Mosaic trisomy 17	Not applicable	Abortion at 22 WG
3	22	Atypical vessel	Hydrocephalus, corpus callosum hypoplasia	Aorta-umbilical vein	Arterial flow	Follow-up	Stationary	Normal	Not applicable	Abortion at 23 WG
4	13	Atypical vessel	Cystic hygroma PVSA	Aorta-umbilical vein	Arterial flow	Follow-up	Fetal distress	Not performed	Not applicable	Abortion
5	13	Atypical vessel	Bilateral radius agenesis	Aorto-hepatic vein	Not evaluated	Follow-up	Stationary	T18	Not applicable	Abortion
6	13	Atypical vessel	Megacystis, hand and foot malposition, skeletal malformations, hygroma, hemivertebra, VSD absent CSP	Aorta-umbilical vein	Not evaluated	Follow-up	Fetal distress	Not performed	Not applicable	Abortion
7	13 Twin pregnancy	Atypical vessel	Absent	Aorta-umbilical vein	Arterial flow	Follow-up	Fetal distress	T21	Not applicable	IUD of the affected fetus
8	30	Dilated vascular channels within the liver	Dilation of right heart chambers	Aorto-hepatic vein	Arterial flow	Follow-up	Stationary	Normal	Embolization of the fistulous tract	Death due to cardiac failure

IUGR, intrauterine growth restriction; PVSA, portal venous system agenesis; VSD, ventricular septal defect; CSP, cavum septum pellucidum; 45X, Turner Syndrome, T18, trisomy 18; T21, trisomy 21; IUD, intrauterine demise; WG, weeks of gestation.

## Data Availability

The raw data supporting the conclusions of this article will be made available by the authors on request.
